# Principles of Surgery

**Published:** 1891-07

**Authors:** 


### Book Reviews.

PRINCIPLES OF SURGERY. By N. Senn, M. D., Ph. D., Milwaukee, Wis. Prof. of Principles of Surgery and Surgical Pathology in the Rush Medical College, Chicago, Ill.

In the preface of this work, the author sets forth that it is a systematic treatise on the causation, pathology, diagnosis, prognosis and treatment of the injuries and affections which the surgeon is most frequently called upon to treat.

It will be noted that he seeks to connect the modern science of bacteriology more intimately with the etiology and pathology of surgical affections than has heretofore been done by most authors who have written on the same subjects.

The book is based upon a recognition, to its fullest extent, of the germ theory of the origin of abnormal processes in the organization, and those who do not accept this, in all its bearings, may conclude that undue weight has been given to the use of so-called antiseptic solutions to recent wounds. It is stated, for instance, that accidental wounds must always be considered as infected wounds, and a faithful effort must be made to render them aseptic by exposing, if possible, the entire wounded surface to the direct action of one of those solutions, including a 1 to 1000 solution of corrosive sublimate.

With the lights before the profession, as to the favorable issue of operating in normal structions, and the closure of clean cuts in healthy tissues without the use of such antiseptic washes, this position cannot be sustained by the testimony of facts. When it is further considered that numerous instances are on record of the injurious constitutional influence of the absorption of corrosive sublimate from local applications of the recognized solutions of it, to fresh incisions, the surgeon should exercise the greatest caution in its use. If there is no good reason to suspect septic contamination the practitioner will be at a loss to understand why "antiseptic precautions are employed for the purpose of securing for the wound and everything that is brought in contact with it, an aseptic condition."

On the other hand, in the treatment of a septic wound, all will concur in the statement of the author, that "no time should be lost in scuring for the wound and its vicinity, an aseptic condition by thorough disinfection."

Under the general head of regeneration of different tissue, it is claimed that no positive proof has yet been furnished that the leucocytes or any other of the cellular elements of the blood take any active part in the restoration of lost parts.

The elements which enter into the organization are enumerated, with their special characteristics, viz: Non-vascular tissue, vascular tissue, connective tissue, muscles, bone and nerves.

Inflammation is given a prominent place, and it is held that this term in the future should be limited to the series of histological changes, which arise in the living body from the presence and action of specific micro-organisms. The old division into acute, subacute and chronic, is adopted, based upon the intensity of symptoms, and the time required to reach one of its terminations.

In the treatment of inflammation, the author maintains his consistency by recommending the saturation of the inflammed tissues with germicidal solutions.

A thorough disquisition upon pathogenic bacteria is in strict conformity to the plan of the work, and it is claimed that in all infective processes in which life is not destroyed, and the products of inflammation do not find their way to the surface spontaneously or by operative treatment, the microbes are either destroyed in the blood and the tissues by phagocytosis or are eliminated through some of the existing organs in an active state.

The several topics of a more practical nature, such as Necrosis, Suppuration, Septicemia, Pyaemia, Erysipelas, Tetanus and Hydrophobia are disposed of in a manner that will prove more acceptable to the practitioner. But it is feared that the etiological and pathological explanations may prove too erudite for most readers.

In the differential diagnosis of furuncle, carbuncle and malignant pustule, it is stated that a furuncle presents only one center of suppuration, is more circumscribed, more superficial and not attended with such marked infiltration as carbuncle.

Malignant pustule is primarily not a suppurating lesion, as it is caused by the bacillus of anthrax, and develops from one point of infection and gives rise to necrosis of the skin at an early age. Carbuncle starts simultaneously or in rapid succession, from three to a dozen or more suppurating foci, is attended by a hard condition of the surrounding connective tissue and gives rise always to multiple foci of necrosis of the subacutenous connective tissue.

The professiou should feel under obligations for this clearing up of the muddle which has existed from the confusion of the term carbuncle and anthrax in our Text Books; and only a few words could be added in elucidation of the various local measures used in these widely different affections. The reviewer's experience with lunar costic and adhesive plaster in arresting carbuncle by inducing prompt suppuration, meets all the requirements in a large majority of cases. During the stage of localization in malignant pustule a free use of the actual cautery for the complete destruction of the papillary development is comparatively painless and has proved efficacious in preventing the secondary constitutional disturbance with its given accompaniments and fatal results.

While there are many other features of great interest to the surgical student and philosopher in this elaborate treatise, it is doubtful whether it adequately meets the demands of this utilitarian and practical age in the bacteriological explanation of all surgical diseases. Many will profit, however, by its teachings. J. Mc. F. G.

---

FARADIZATION IN INCONTINENCE OF URINE.—Dr. Jamin reports a case of incontinence in a girl aged fifteen, where internal medication had failed of any result, in which a complete cure was obtained by faradization of the urethra—the negative pole in the urethra, and the positive on the thigh.—*Med. Record.*

---

The prohibition of the sale of tuberculin in Munich has now been made absolute. Druggists are forbidden to sell it even to medical men.—*Ex.*

